# Cytopathology and ultrastructure identification of primary hepatic acinar cell carcinoma: Case report

**DOI:** 10.1016/j.ijscr.2019.08.006

**Published:** 2019-08-17

**Authors:** J.G. Grab, D. Skubleny, N.M. Kneteman

**Affiliations:** Faculty of Medicine and Dentistry, University of Alberta, Department of Surgery, Division of General Surgery, University of Alberta Hospital 8440 - 112 Street, Edmonton, Alberta T6G 2B7, Canada

**Keywords:** Acinar cell carcinoma, Electron microscopy, Imaging, Cytopathology, Hepatobiliary surgery

## Abstract

**Introduction:**

A primary acinar cell carcinoma (ACC) of the liver was incidentally diagnosed in a clinically asymptomatic 80-year-old man. This study aimed to delineate critical diagnostic characteristics of an ACC originating uniquely from the liver to improve its future identification.

**Presentation of case:**

Enhanced MRI revealed a heterogenous, cystic 7.7 × 11.1 × 10.4 cm tumour occupying hepatic segments II and III. The mass demonstrated mild diffuse enhancement in hepatic arterial phase with minimal portal venous washout in a liver without cirrhotic features. A central stellate T2-hyperintense necrotic scar and outer capsule were apparent. No primary lesion or metastasis outside the liver was discernable. Post-left hepatic lobectomy, the tumour immunophenotype was atypical for presumptive diagnoses of hepatocellular carcinoma (HCC) or cholangiocarcinoma. Extensive morphologic workup on electron microscopy definitively diagnosed primary hepatic ACC by establishing presence of secretory zymogen-like granules, intracytoplasmic microvilli and acinar cell differentiation. Cytopathology revealed cellular lumen expressing PAS-positive diastase-resistant granular cytoplasmic contents.

**Discussion:**

This case showcased the novel utility of electron microscopy that was crucial in yielding the definitive diagnosis. The previous literature on hepatic ACC was compiled here in context of the present case. The mechanism of hepatic acinar cell localization was also discussed.

**Conclusion:**

Primary hepatic ACC may easily be confused for other lesions due to nonspecific imaging patterns. Specifically, the presence of a central scar without risk factors for HCC can favour a diagnosis of benign entities such as focal nodular hyperplasia (FNH). Electron microscopy presents an important tool to identify primary hepatic ACC and may improve future patient outcomes.

## Highlights

• Acinar cell carcinoma originating in the liver proper remains a diagnostic quandary.
• Non-specific imaging features may have led to prior misdiagnosis and poor outcomes.
• Ultrastructural electron microscopy represents a novel tool for identification.
• Heterotopia or metaplastic mechanisms may underlie hepatic acinar cell localization.
• The previous literature is summarized in context of the present case of hepatic ACC.

## 1 Introduction

Acinar cell carcinoma (ACC) is a rare malignancy that is typically localized to the pancreas or salivary glands. Approximately 98% of pancreatic tissue is composed of acinar cells which aid in pancreatic exocrine function [1], however despite the abundance of this cell type, malignant transformation is uncommon. Overall, ACC represents 1–2% of adult malignancies [2]. Despite the wealth of data on pancreatic and salivary ACC in the literature, studies characterizing ACC arising in non-typical regions have not been overly documented. Most cases relate to malignant transformation and acinar differentiation of ectopic pancreatic tissue [[3], [4], [5]].

Only a handful of documented cases report ACC originating in the liver, the first of which was documented in 2008 by Hervieu and colleagues [6]. ACC of hepatic origin has been sparingly studied since its recognition. A clinicopathologic study of 4 patients [7], magnetic resonance imaging (MRI) findings [8], and two case reports of multimodal surgical and chemotherapeutic regimens [9,10] have been documented previously.

To date, no report has demonstrated the utility of electron microscopy in establishing a diagnosis of primary hepatic ACC. It is evident that primary hepatic ACC remains an elusive diagnostic quandary where correct and early diagnosis may avoid a delay in treatment, guide targeted management, and improve patient outcomes. We report a case of primary hepatic ACC to further delineate a focused diagnostic workup surrounding the ultrastructural morphologic and molecular characteristics of this tumour. The aim of this study is to assist in future recognition of this rare disease and compile the previous case reports on primary hepatic ACC.

This work is in accordance with the Surgery CAse Report (SCARE) consensus guidelines [11].

## 2 Presentation of case

An 80-year-old Caucasian man presented in December 2017 to our institution after incidental imaging discovered a large tumour in liver segments II and III. The patient endorsed chronic progressive fatigue but denied abdominal pain or other constitutional symptoms of weight loss, fevers or night sweats. The past medical history was significant for multiple comorbid illnesses including: dilated cardiomyopathy with an ejection fraction of 40%, atrial fibrillation, hypertension, and history of deep vein thrombosis requiring lifelong warfarin anticoagulation. Also affecting this patient was an unknown interstitial nephritis that required a short duration of hemodialysis, monoclonal T-cell gammopathy, cholelithiasis, mild chronic obstructive pulmonary disease (COPD) as well as an unknown primary eosinophilic hematologic disease requiring treatment with azathioprine. There was a history of cigarette smoking of a pack per day for 35–40 years, however the patient had quit 20 years prior. Physical examination revealed a soft yet focally distended abdomen and a firm, nontender mass localized to the epigastrium.

**Fig. 1:**
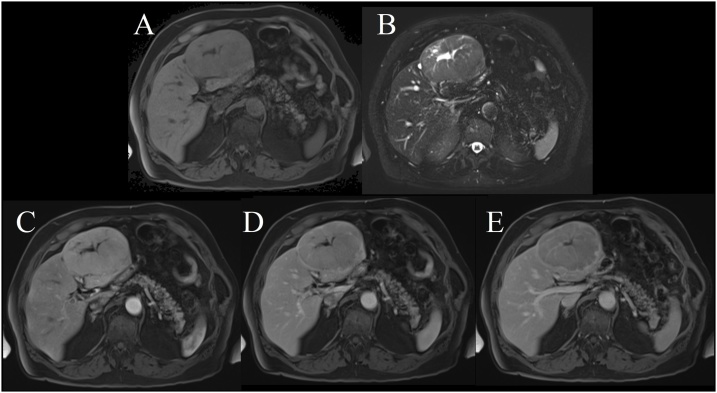
Primary hepatic ACC focal to segments 2 and 3. T1-weighted images shown as A, while T2-weighted images shown in B. The mass shows mild hypointensity, mild T2-weighted hyperintensity and restricted diffusion. Within it, a central stellate area of T1-weighted hypointensity and T2-weighted hyperintensity is visible. The internal central "scar" does not enhance. Within the mass, some radiating enhancing septae are visible. There is no fat nor lipid hyperexpression within the mass. The mass does exhibit mild diffuse enhancement in hepatic arterial phase (A – pre contrast; C – 0.5 min post contrast; D – 1 min post contrast) with slight portal venous phase washout and, on delayed imaging, a capsule (E – 5 min post contrast).

Laboratory analysis only showed a mild elevation of GGT (111–124). Cancer markers including AFP and CEA were negative. A large, smooth, focal and well-circumscribed 7.7 × 11.1 x 10.4 cm mass (AP x transverse x cephalocaudal maximal dimensions) with septations and an internal central scar was visualized on gadolinium-enhanced MRI of the patient’s liver (Fig. 1). The mass was not apparent on abdominal MRI or ultrasound just 19 months prior to the incidental discovery. The imaging characteristics on T1-weighted imaging included mild diffuse hepatic arterial enhancement, minimal portal venous washout and a clearly visible capsule that all strongly suggested fibrolamellar hepatocellular carcinoma (HCC). FNH was also made a possibility given a hyperintense central scar on T2-weighted imaging. A splenule identified as early as 5 years prior, was stable in size with features inconsistent for a possible primary lesion.

**Fig. 2:**
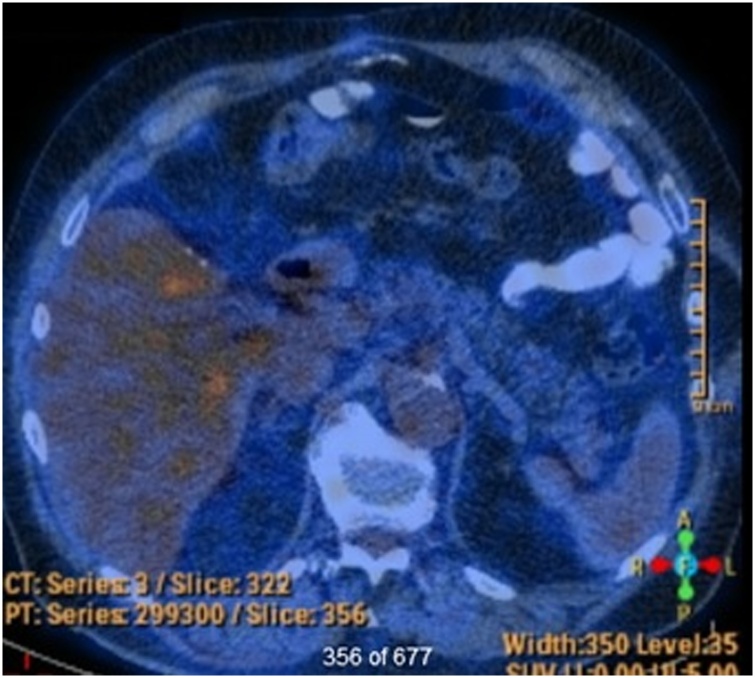
Positron emission tomography (PET) scan status 10 months post left hepatic lobectomy. No evidence of FDG avid local or distant metastatic disease was apparent.

The patient underwent a left hepatic lobectomy and open cholecystectomy. Intraoperatively, the parenchymal transection was noted to be “bloodier than usual due to very fragile veins that were hard to dissect before they were already bleeding.” The patient recovered well post-operatively without operative complications. An interdisciplinary team decision to not offer adjuvant chemotherapy was made given the unclear benefit and the patient’s significant risk of deterioration. No local-regional or distant disease was identified on positron emission tomography (PET) scanning 3 and 10 months post-operatively (Fig. 2). The aforementioned splenule was again detected unchanged and equivalent in FDG avidity as the spleen. Case report presentations, imaging characteristics, and differential diagnoses of primary hepatic ACC in the literature are presented in Table 1.

**Table 1:** Case presentations, defining characteristics, differential diagnoses, as well as outcome of the current study and five previous case reports of hepatic acinar cell carcinoma.

Study	Age (Years)	Gender	Presentation	Comorbid Disease	Laboratory Tests	Defining Characteristics	DDX on Imaging	Definitive Diagnosis	Treatment	Follow-Up Post Diagnosis
Current Study	80	M	Asymptomatic	Multiple (See Case Study Report)	Within normal limits	7.7 × 11.1 × 10.4 cm Mass Heterogenous, Septated, Central Scar, Encapsulated, Hypoenhancement (Enhanced MRI)	- Fibrolamellar HCC - Cholangiocarcinoma	Pathology	Formal Resection	No Disease at 10 Months
Laino et al (2018)	48	M	Obstructive Jaundice	None Reported	Obstructive Biliary Pattern	14 × 15 cm Mass Heterogenous, Cystic, Septated, Encapsulated, Delayed Enhancement, No Capsule (Enhanced CT)	- Fibrolamellar HCC - Cholangiocarcinoma - Angiosarcoma - Epithelioid Hemangioma - Hepatoblastoma - Distant Metastases	Pathology	Palliative Chemotherapy (Xeloda/ Oxaliplatin)	No Disease at 13 Months
Jordan et al (2017)	54	F	None	None Reported	Transaminitis, AFP of 82 IU/mL	Two Masses: 12.9 × 10.4 × 14.2 cm and 3.0 × 2.8 cm Hypoenhancment (Enhanced MRI)	- Not Disclosed	Pathology	Formal Resection, Smaller Mass Unresectable Palliative Chemotherapy (FOLFOX)	Shrinking Local-Regional Disease at 20 Months
Wildgruber et al (2013)	31	M	Abdominal Pain	None Reported	Within normal limits	Heterogenous, Central Scar, Strong Arterial Blush (Enhanced MRI and CT)	- Fibrolamellar HCC - Hemangioendothelioma - Angiosarcoma - Cholangiocarcinoma	Pathology	Palliative Chemotherapy (Not Reported)	Died at 18 Months
Agaimy et al (2011)	68	F	Non-Specific Symptoms	None Reported	Within Normal Limits	7–19 cm (mean of 12 cm) Heterogenous, Cystic, Septated, Encapsulated, Central Necrotic Scar, Focal Hemorrhage (Unenhanced CT)	- Fibrolamellar HCC - Cholangiocarcinoma	Pathology	Formal Resection	No Disease at 38 Months
	71	M							Formal Resection	Died at 3 Months
	72	M							Formal Resection Recurrence with Chemotherapy	Local Recurrent Disease at 22 Months
	49	F							Formal Resection	No Disease at 28 Months
Hervieu et al (2008)	35	F	Abdominal Pain, Constitutional Symptoms	Hepatitis B	AFP of 6000 IU/mL	5 × 5 cm Well limited, No Capsule, Hypervascular (Unenhanced CT)	- Fibrolamellar HCC	Pathology	Formal Resection	No Disease at 7 Years

**Fig. 3:**
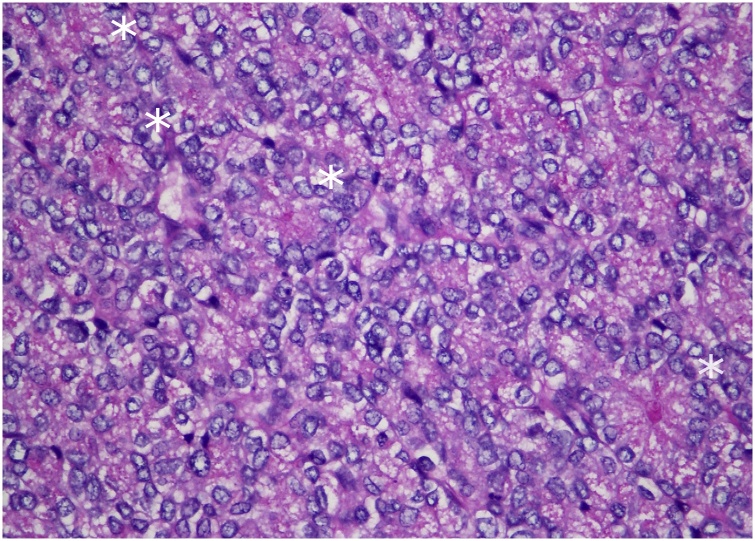
Diastase-digested periodic acid-Schiff (PAS) stain shows intracellular PAS-positive granules consistent with secretory granules. Focally, microacinar structures (white asterisks) are visible. Original magnification x400.

**Fig. 4:**
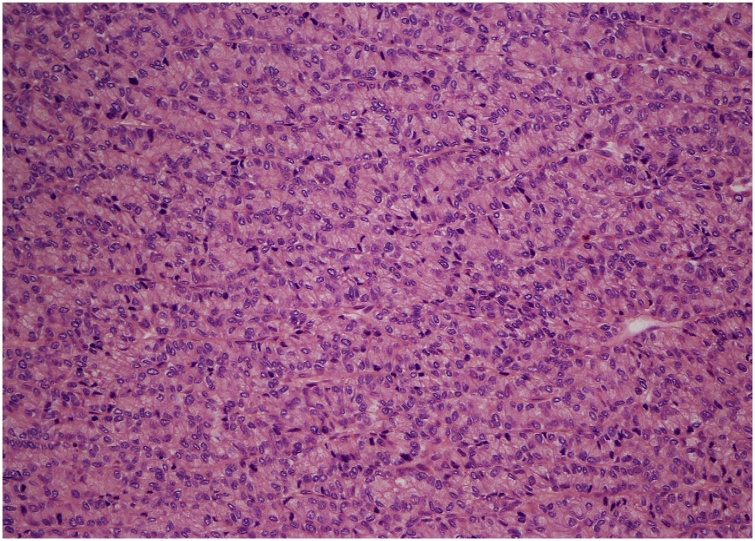
Tumour with anastomosing acinar and trabecular growth pattern. Hematoxylin-eosin, original magnification x200.

The preliminary pathology assessment was of a large tumour with negative margins that occupied 95% of the noncirrhotic left hepatic lobe. The tumour was made up of nested rosette-like acinar structures with elongated compressed cord-like vessels in between. Cells had fine granular eosinophilic cytoplasm, pale ovoid nuclei with nuclear grooves, and infrequent mitoses (Fig. 3). Anastomosing acinar and trabeculated growth patterns were also observed (Fig. 4).

Consistent with a rapidly growing tumour, 15% of MIB-1 cells were identified. The immunophenotype was atypical for HCC as there was no expression of HepPar1 or AFP with moderate expression of cytokeratin 7. Furthermore, ruling out cholangiocarcinoma, true gland-like structures with a developed lumen were not present in conjunction with a negative cytokeratin 19 (Table 2).

**Table 2:** Immunostaining selectivity of five previous primary hepatic acinar cell carcinoma (ACC).

Primary Hepatic ACC Literature						
Tumour Marker	Current Study	Laino et al (2018)	Jordan et al (2017)	Wildgruber et al. (2013)	Agaimy et al. (2011)	Hervieu et al. (2008)
LMW keratin	+					
CK7	+				–	+
CK18					+	
CK19	–		+		–	
CK20	–				–	–
CD10	–				–	
CD31	–					
CD45	–					
CD56 (N-CAM)	–				–	
CDX2					–	
Synaptophysin	–		+		+	+
Chromogranin A	–		+		+	+
HepPar-1	–		–		–	+
AFP	–				–	+
MOC31	+					
GATA3	–					
DOG1	–					
TTF1	–		–			
S-100	–					
HMB45	–					
MIB-1	+ (15%)				+ (2-10%)	+ (5-15%)
Trypsin		+	+		+	+
Chymotrypsin			+		+	
Amylase			–		+	
Lipase			–		+	
ER			–			
PR			–			
KL-1					+	
Polyclonal CEA					–	
α1-AT						+
E-Cadherin						+
β-Catenin						+

**Fig. 5:**
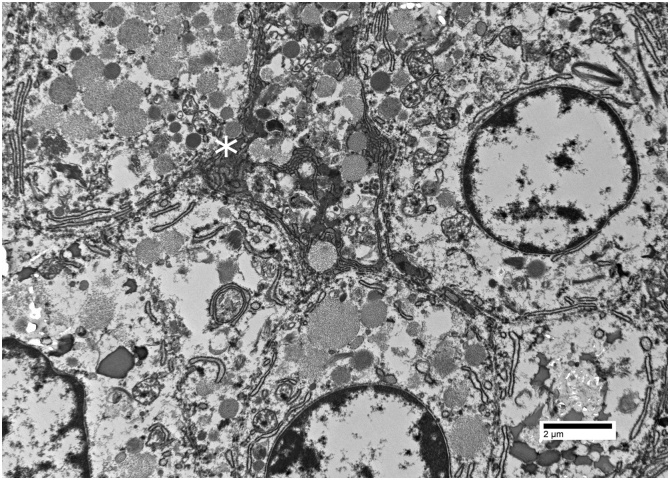
Electron micrograph showing intracytoplasmic microvilli as well as intracytoplasmic membrane-bound vesicles (asterisk) consistent with secretory zymogen granules. Direct magnification x2500.

After light microscopy and immunostaining, the main differential included cholangiocarcinoma, HCC with microacinar pattern or primary hepatic ACC. On electron microscopy, round to polygonal cells contained numerous membrane-bound vesicles consistent with zymogen granules. Irregular filamentous bundles were identified and similar to that previous described for pancreatic ACC [12]. Occasional cells also demonstrated intracytoplasmic microvilli which indicated epithelial specialization (Fig. 5).

## 3 Discussion

Acinar cell carcinoma is a rare malignant neoplasm that may arise in uncommon locations such as in this case of a primary lesion confined to the liver. Patients with primary hepatic ACC typically present late either from incidental imaging or due to work-up of nonspecific symptoms. Up to 50% of patients may have metastatic disease at initial discovery [7]. Furthermore, current imaging modalities alone are insufficient for definitive diagnosis. Primary hepatic ACC may easily be confused for other malignant tumours such as HCC, cholangiocarcinoma, hypervascular metastases [7,10] or misdiagnosed as benign entities like focal nodular hyperplasia (FNH) and hemangioma. Improper identification may therefore lead to absent or delayed treatment. This fact is reflected in the key imaging features of classic FNH that resemble hepatic ACC which include a homogenously iso-intense lesion with central scar on T2-weighted imaging. This pattern represents 80% of FNH cases where upon imaging recognition, if asymptomatic, no further treatment is necessary [13].

Ultrastructural determination of primary hepatic ACC with electron microscopy in conjunction with immunohistochemical staining proved to be an invaluable adjunct for definitive diagnosis. Diagnostic criteria of primary hepatic ACC should include electron microscopy where available and is provided as followed: 1) acinar cell type morphology and architecture with a lumen containing periodic acid-Schiff (PAS)-positive secretion. 2) electron microscopic confirmation of secretory zymogen granules 3) immunohistochemistry demonstrating negative staining for liver cell types or HCC (negative HepPar1), for bile duct epithelium or cholangiocarcinoma (negative cytokeratin 19), or neuroendocrine tumour (negative Chromogranin and Synaptophysin). 4) Presence of positive stains for trypsin, chymotrypsin, or amylase [8].

The mechanism in which acinar cells became localized to the liver and undergo malignant transformation remains unknown. One proposed mechanism of localization may be due to anomalous development and heterotopic displacement of pancreatic progenitors to the developing liver. The highest chance of occurrence would be the fourth week of embryologic development where the ventral pancreatic bud and hepatic diverticulum exist spatially adjacent to one another as outgrowths of the caudal portion of the foregut. Evidence for this mechanism was demonstrated by Terada and colleagues [14] where human post-mortem examinations were conducted to determine the proportion of specimens with intrahepatic heterotopic pancreatic tissue [14]. Of the 1000 specimens assessed, pancreatic acini-type differentiation was positive in 4.1% of livers lending to the anomalous development/heterotopia hypothesis.

A second candidate mechanism involved trans-differentiation of liver into acini-type pancreatic tissue. Kuo and colleagues examined human liver explants for the presence of pancreatic-type tissue. Similar to the post-mortem study by Terada and colleagues, 4.2% of explants contained pancreatic acini-type differentiated tissue [14,15]. However, in positive liver explants for acinar tissue, the majority had been exposed to known *in vivo* inflammatory insults caused by viral hepatitis, HCC or cholangitis. It was surmised that pancreatic acini in liver explants were the result of metaplasia of hepatic progenitor cells through a reactive bile duct intermediary. Evidence supporting this metaplastic hypothesis included near identical immunohistochemical staining profiles between pancreatic acini-type tissue and the closely residing adjacent bile ductules (positive staining for chromogranin A, CK7, CK8, and CK19 with reactive CD56 positive) [15]. This profile was also interestingly obtained from differentiating hepatic progenitor cells in the liver explants. Notably, there was also an appreciable lack of detectable endocrine cells or islets of Langerhans which contradicted a heterotopic mechanism, lending to a metaplastic mechanism underlying acinar cell identification within the liver.

## 4 Conclusion

Primary hepatic ACC is a diagnosis that requires consideration. Specifically, masses with a central scar and MRI intensity patterns typical to more common lesions (HCC, cholangiocarcinoma, or FNH) should not be misidentified and may require more extensive workup to fully elucidate. Only five other reports of primary hepatic acinar cell carcinoma exist in the literature where pathology was essential in definitive diagnosis. Here, this report showcases the novel utility of electron microscopy for yielding ultrastructural diagnosis. This case represents an important identification of primary hepatic ACC with review of the previous cases to help aid future diagnosis and improve patient outcomes.

## Funding

No grant funding was associated in creation of this manuscript.

## Ethical approval

The Research Ethics Office (REO) at the University of Alberta has given our case report exemption status.

## Consent

Written and signed informed consent was obtained by the patient for publication and accompanying images prior to creation of this manuscript and collection of personal information. A copy of this consent is available for review by the Editor-in-Chief of the journal on request.

## Registration of research studies

UIN is retrievable on researchregistry.com with the UIN of researchregistry5028.

## Guarantor

The guarantor is Dr Norman M Kneteman.

## Provenance and peer review

Not commissioned, externally peer-reviewed.

## CRediT authorship contribution statement

**J.G. Grab:** Conceptualization, Data curation, Formal analysis, Validation, Visualization, Writing - original draft, Writing - review & editing. **D. Skubleny:** Conceptualization, Formal analysis, Project administration, Supervision, Writing - review & editing. **N.M. Kneteman:** Conceptualization, Investigation, Methodology, Project administration, Resources, Supervision, Validation, Visualization, Writing - review & editing.

## Declaration of Competing Interest

There are no conflict of interests to disclose.

## References

[1] Guney M.A., Gannon M. (2009). Pancreas cell fate. Birth Defects Res. Part C Embryo Today Rev..

[2] Wood L.D., Klimstra D.S. (2014). Pathology and genetics of pancreatic neoplasms with acinar differentiation. Semin. Diagn. Pathol..

[3] Cingolani N., Shaco-Levy R., Farruggio A., Klimstra D.S., Rosai J. (2000). Alpha-fetoprotein production by pancreatic tumors exhibiting acinar cell differentiation: study of five cases, one arising in a mediastinal teratoma. Hum. Pathol..

[4] Moncur J.T., Lacy B.E., Longnecker D.S. (2002). Mixed acinar-endocrine carcinoma arising in the ampulla of vater. Hum. Pathol..

[5] Mizuno Y., Sumi Y., Nachi S., Ito Y., Marui T., Saji S., Matsutomo H. (2007). Acinar cell carcinoma arising from an ectopic pancreas. Surg. Today.

[6] Hervieu V., Lombard-Bohas C., Dumortier J., Boillot O., Scoazec J.-Y. (2008). Primary acinar cell carcinoma of the liver. Virch. Arch. Int. J. Pathol..

[7] Agaimy A., Kaiser A., Becker K., Bräsen J.-H., Wünsch P.H., Adsay N.V., Klöppel G. (2011). Pancreatic-type acinar cell carcinoma of the liver: a clinicopathologic study of four patients. Mod. Pathol. Off. J. U. S. Can. Acad. Pathol. Inc..

[8] Wildgruber M., Rummeny E.-J., Gaa J. (2013). Primary acinar cell carcinoma of the liver. ROFO Fortschr. Geb. Rontgenstr. Nuklearmed..

[9] Jordan E.J., Basturk O., Shia J., Klimstra D.S., Alago W., D’Angelica M.I., Abou-Alfa G.K., O’Reilly E.M., Lowery M.A. (2017). Case report: primary acinar cell carcinoma of the liver treated with multimodality therapy. J. Gastrointest. Oncol..

[10] Laino M.E., Ragucci M., Klimstra D.S., Mannelli L. (2018). Hepatobiliary and pancreatic: primary acinar cell carcinoma of the liver showing good response to chemotherapy. J. Gastroenterol. Hepatol..

[11] Agha R.A., Borrelli M.R., Farwana R., Koshy K., Fowler A.J., Orgill D.P., SCARE Group (2018). The SCARE 2018 statement: updating consensus Surgical CAse REport (SCARE) guidelines. Int. J. Surg. Lond. Engl..

[12] Klimstra D.S., Heffess C.S., Oertel J.E., Rosai J. (1992). Acinar cell carcinoma of the pancreas. A clinicopathologic study of 28 cases. Am. J. Surg. Pathol..

[13] Hussain S.M., Terkivatan T., Zondervan P.E., Lanjouw E., de Rave S., IJzermans J.N.M., de Man R.A. (2004). Focal nodular hyperplasia: findings at state-of-the-art MR imaging, US, CT, and pathologic analysis. RadioGraphics.

[14] Terada T., Nakanuma Y., Kakita A. (1990). Pathologic observations of intrahepatic peribiliary glands in 1000 consecutive autopsy livers. Heterotopic pancreas in the liver. Gastroenterology.

[15] Kuo F.-Y., Swanson P.E., Yeh M.M. (2009). Pancreatic acinar tissue in liver explants: a morphologic and immunohistochemical study. Am. J. Surg. Pathol..

